# Book Reviews

**Published:** 1895-07

**Authors:** 


### Book Reviews.

Year-Book of Treatment. Philadelphia, Pa. Lea Bros. & Co., 1895.

The Year-Book of Treatment for 1895 gives an excellent epitome of treatment of diseases in all provinces of medicines. The work is admirably suited to those who desire to gain condensed information without waste of time and unnecessary amount of reading. The chapter on Bacteriology is missing but its loss is not conspicuous in a practical work of this kind.

The Care of the Baby. A Manual for Mothers and Nurses. By J. Crozer Griffith. W. B. Saunders, Philadelphia, 1895. Price, $1 50.

The Care of the Baby, by J. P. Crozer Griffith, is a book intended as a guide to nurses and young mothers, and it will certainly give the latter much better advice than that so freely offered by well-meaning but insufficiently informed friends. It contains well written chapters on the management, diet and commoner diseases of the newly born, together with the management of the mother during the puerperium. The work is well illustrated throughout.

International Clinics, Vol. I., Fifth Series. J. B. Lippincott & Co., Philadelphia, Pa.

International Clinics is of especial value to the general practitioner of medicine and surgery as showing the direct application of the principles of treatment to *the*, not *a* patient. The work is thus rendered much more impressive and instructive than the generalization of text-books. The patient is brought so closely and clearly before the mind of the reader that he can almost see him and hear the professor's expounding voice, and at the end he has a full and intelligent understanding of the subject and can apply what he has learned to cases in his own practice. The series can most heartily be recommended.

Transactions of the Southern Surgical and Gynæcological. Association—Vol. VII, 1894. Printed by the Association.

Transactions of the New York Academy of Medicine, Second Series, 1893. Vol. X. Printed for the Academy, 1894.

Some Points in the Technique of Kidney Operation. By Chas. S. Briggs, A. M., M. D., Nashville, Tenn.

Phelps' Operation for Club-foot. By C. S. Briggs, A. M., M. D., Nashville, Tenn.

Scopolamine As a Mydriatic. By Arthur G. Hobbs M. D., Atlanta.

When to Wear Glasses and How to Choose Them. By Arthur G. Hobbs, M. D., Atlanta.

Urinalysis, including Blanks for Recording Analysis and Microscopic Examination of the urine. For Medical Practitioners, Life Insurance Examiners and Specialists. Arranged by Joseph C. Guernsey, A. M., M. D. J. B. Lippincott, Philadelphia.

